# Potential association factors for developing effective peptide-based cancer vaccines

**DOI:** 10.3389/fimmu.2022.931612

**Published:** 2022-07-27

**Authors:** Chongming Jiang, Jianrong Li, Wei Zhang, Zhenkun Zhuang, Geng Liu, Wei Hong, Bo Li, Xiuqing Zhang, Cheng-Chi Chao

**Affiliations:** ^1^ Department of Medicine, Baylor College of Medicine, Houston TX, United States; ^2^ Dan L Duncan Comprehensive Cancer Center, Baylor College of Medicine, Houston, TX, United States; ^3^ Institute for Clinical and Translational Research, Baylor College of Medicine, Houston, TX, United States; ^4^ Institute of Super Cell, BGI-Shenzhen, Shenzhen, China; ^5^ Department of Pipeline Development, Biomap, Inc, San Francisco, CA, United States

**Keywords:** cancer vaccine, peptide, clinical response, machine learning, potential factors

## Abstract

Peptide-based cancer vaccines have been shown to boost immune systems to kill tumor cells in cancer patients. However, designing an effective T cell epitope peptide-based cancer vaccine still remains a challenge and is a major hurdle for the application of cancer vaccines. In this study, we constructed for the first time a library of peptide-based cancer vaccines and their clinical attributes, named CancerVaccine (https://peptidecancervaccine.weebly.com/). To investigate the association factors that influence the effectiveness of cancer vaccines, these peptide-based cancer vaccines were classified into high (HCR) and low (LCR) clinical responses based on their clinical efficacy. Our study highlights that modified peptides derived from artificially modified proteins are suitable as cancer vaccines, especially for melanoma. It may be possible to advance cancer vaccines by screening for HLA class II affinity peptides may be an effective therapeutic strategy. In addition, the treatment regimen has the potential to influence the clinical response of a cancer vaccine, and Montanide ISA-51 might be an effective adjuvant. Finally, we constructed a high sensitivity and specificity machine learning model to assist in designing peptide-based cancer vaccines capable of providing high clinical responses. Together, our findings illustrate that a high clinical response following peptide-based cancer vaccination is correlated with the right type of peptide, the appropriate adjuvant, and a matched HLA allele, as well as an appropriate treatment regimen. This study would allow for enhanced development of cancer vaccines.

## Introduction

Cancer is a heterogeneous disease with mixed clinical outcomes (1). Conventional cancer treatments tend to non-specifically kill tumor cells. Some of these tumor cells survive due to resistance to therapy and drug toxicity, eventually leading to tumor relapse and metastasis (2, 3). Cancer immunotherapy is a treatment strategy that uses a patient’s own immune system to fight the cancerous cells (4, 5). Immunotherapy has become a promising alternative cancer treatment after surgery, radiotherapy, and chemotherapy in recent years because of its mild side effects and significant therapeutic benefits (6–8). T cell epitope peptide-based cancer vaccine is one of the representative strategies of cancer immunotherapy, relying on short peptide fragments to engineer the induction of highly targeted immune responses (9–11).

Previous studies (12–17) have demonstrated the effectiveness of peptide-based cancer vaccines in treating several common types of cancer, such as breast cancer, melanoma, colorectal and lung cancer. This strategy exploits the fact that the surfaces of cancer cells have various peptide epitopes (i.e., peptides of usually 8-17 residues in length), which bind to major histocompatibility complex (MHC) proteins. T cells can attack these cancer cells after recognizing the peptide/MHC complex (18). T cells aimed to induce immune recognition of tumor cells are then able to eradicate them by generating a sustained and potent anti-tumor immune response. Therefore, a key determinant for an anti-tumor immune response to lead to the effective killing of cancer cells is the selection of immunogenic peptide epitopes as the target (19). Many peptide epitopes have been identified and molecularly characterized in experiments (12–17). While there are many options in selecting immunogenic antigens, it is not clear which selected epitopes can induce the dominant immune system mediated by T cells. Many clinical studies in cancer vaccines have been initiated to assess the therapeutic potential of active immunization or vaccination with peptide epitopes in cancer patients. However, it is still unclear what the ideal characteristics of selected peptide epitopes should be and which could induce stronger anti-tumor responses. Therefore, it remains highly challenging to design an effective cancer vaccine that can achieve a meaningful clinical benefit in patients.

There have been many breakthroughs in prior studies that investigated the optimal conditions for designing a peptide-based cancer vaccine (20–25). Thomas et al, Zhang et al, and Liu et al found differences in the therapeutic efficacy of peptide-based cancer vaccines prepared from different sources of peptides (26–28). Furthermore, patients with certain HLA alleles might be more sensitive to respond to cancer vaccines (29, 30). The same cancer vaccine with different adjuvants might also have an impact on the outcome of treatment (28, 31). In addition, different treatment strategies could also affect the efficacy and side effects of cancer vaccines, such as the dose of vaccine used, injection interval, number of injections, and injection methods (28, 32). However, due to the limited number of clinical trials available, combined with the difference in cancer types and patient conditions, it is difficult to improve a cancer vaccine design by directly referring to the design of other cancer vaccines. Machine learning (ML) options such as Random forest (RF) (33)could improve cancer vaccine design by utilizing large data repositories to identify novel features and more complex interactions among these features.

In this study, a library of peptide-based cancer vaccines used in clinical studies from public and private sources was established from multiple sources, such as PubMed, ClinicalTrials.gov, and Web of Science, using databases up to January 1, 2022. The statistical analysis of types of peptides, adjuvants, treatment regimen, human leukocyte antigen (HLA) alleles of peptides, and other features in vaccine therapy was obtained from the results in high clinical response (HCR) and low clinical response (LCR) in the database to find the associations which influence the treatment effect of cancer immunotherapy. Finally, we built a random forest model to help distinguish which kinds of cancer vaccines in patients are most likely to achieve a high clinical response.

## Material and methods

### Data utilized in this study screening and extraction

We screen and extracted all the peptide-based cancer vaccine relevant studies, retrieved from the PubMed, ClinicalTrials.gov, and Web of Science, using databases up to January 1, 2022. All studies were browsed, searched, prioritized, and filtered by the investigators based on the keywords: peptide*, vaccine*, cancer/tumor*, human, HLA, clinical. These extracted studies were then reviewed according to the inclusion and exclusion criteria. In cases in which the results obtained were different, the case was discussed further to obtain consensus. Further details are provided in the following sections. Finally, A total of 705 peptides resulting from 152 clinical studies containing peptide-based cancer vaccine features and clinical treatment information were registered in our library, which has been posted to our web-accessible library, CancerVaccine (https://peptidecancervaccine.weebly.com/).

### Inclusion criteria

The inclusion criteria were as follows: 1. The study focused on human cancer research. 2. The study used the peptide as the vaccine to treat cancer patients. 3. They are not review reports; there are cancer detail descriptions and treatment information about the clinical trials.

### Exclusion criteria

The exclusion criteria were as follows: 1. The peptide-based cancer vaccine was not associated with humans. 2. There was no associated peptide information. 3. There was no treatment information for the peptide-based cancer vaccine.

### Feature selection procedure

Exploratory data analysis: First, we checked the types of variables. There were no missing values in the data (**Figure S3A**). Next, we created a bar graph for the categorical variables; if the levels of all categorical variables looked correct, we kept the original levels for these variables (**Figure S3B**). Finally, four features were recommended for the model: injection interval and injection time, adjuvants, and HLA alleles; the blue dot represents the optimal solution, as shown in **Figure S3C**.

### Classifier

We use a random forest model (random Forest package in R) (34) to construct a feature-based classifier. The prediction performance (estimated by 10-fold cross-validation) is best when the top 4 features with the most differentiation are included (injection interval, injection times, adjuvant types, and HLA alleles), indicating that these 4 features have the greatest differentiation power. Using these 4 features as predictors, the area under the receiver operating characteristic (ROC) curve (AUC) was 0.97. The ROC curves were plotted using the pROC R package.

### The area under the precision-recall curve

For computing the AUPRC, we used the function metrics.precision_recall_curve and metrics.auc from the R package, ROCR 1.0-11 version (35). We first randomly divided the library cohort with known high or low clinical response into a training set (50% randomly selected samples) and a test set (50% randomly selected samples) based on cancer type. Then, the merged training set was used as the training set and the merged test set was used as the test set. Finally, we logit-transformed AUPRC values before testing (using two-tailed Welch’s t-test). We carried out 1,000 replications of 5-fold cross-validation; within each replication, across the 5-folds, we obtained prediction scores for each cancer type from the fold in which the cancer type was in the test set, enabling us to compute an overall AUPRC within each replication.

### Statistical analysis

The R statistical package was used for all data processing and statistical analysis (R package: stats v3.6.2). All details of the statistical tests are specified in the associated text or figure legends. For the statistical analyses, P-values were calculated by using the “Wilcox_test” function from the R package: stats v3.6.2, which applies the two-sided Wilcoxon rank-sum test and corrected multiple testing using the Holm–Bonferroni method. A statistically significant difference was assumed when P ≤ 0.05.

## Results

### Data filtering and features of selected trials

**Figure 1:**
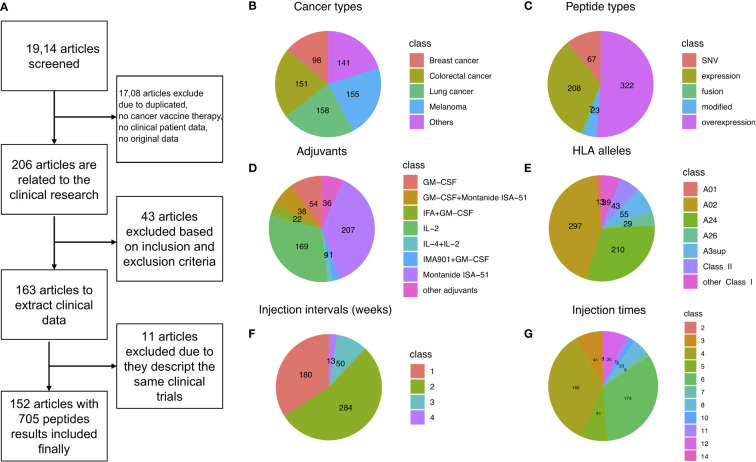
Data filtering summary landscape of the library, CancerVaccine (https://peptidecancervaccine.weebly.com/). **(A)** Data filtering process. **(B)** The landscape of cancer types. **(C)** The landscape of peptide types. **(D)** The landscape of adjuvants. **(E)** The landscape of HLA alleles. **(F)** The landscape of treatment regimen (injection interval). **(G)** The landscape of treatment regimen (injection times).

To investigate what classes of peptide-based cancer vaccine can help achieve satisfactory results from clinical treatment, we reviewed a total of 302 relevant studies, retrieved from the PubMed, ClinicalTrials.gov, and Web of Science, using databases up to January 1, 2022. PubMed.gov is a free search engine that accesses the MEDLINE database on life sciences and biomedical topics primarily at the U.S. National Institutes of Health’s National Library of Medicine. The database of ClinicalTrials.gov is a service of the U.S. National Institute of Health. After removing duplicates, we screened 206 potentially relevant articles by scanning the titles and abstracts. We reviewed the full text and screened the candidate studies according to the inclusion criteria, and 43 studies were excluded. Of the remaining 163 studies, 11 were excluded due to describing the same repeated clinical trials. A total of 705 peptides resulting from 152 clinical studies containing peptide-based cancer vaccine features and clinical treatment information were registered in our library (12–14, 36–163). Details of the study identification process can be found in **Figure 1A**. The final study population included 6,713 participants. All studies were retrospective studies published between January 1, 1997 and January 1, 2022, and involved various tumor types. The details of the library in this study have been posted to our web-accessible library, CancerVaccine (https://peptidecancervaccine.weebly.com/).

In order to analyze this library in a comprehensive and in-depth manner, we mapped the types of cancer and peptide, adjuvant, HLA allele, and treatment regimen (injection interval and injection times) landscapes of the library, as shown in **Figures 1B-G**, respectively. We found that melanoma, colorectal cancer, and breast cancer are the most common cancer types investigated in cancer vaccine therapy (**Figure 1B**). The peptides were divided into five categories based on the origin of peptides: tumor expressed peptides, tumor overexpressed peptides, fusion peptides, modified peptides, and single-nucleotide variant (SNV) peptides. For tumor expressed peptides, the genes in which the peptides were co-expressed are found in both cancer and normal tissues (144, 164–166). For tumor overexpressed peptides, the genes in which the peptides were located are found overexpressed in the tumor tissue only (129, 167, 168). For fusion peptides, the peptides were derived from gene fusion (57, 122, 130, 136, 155). For modified peptides, the amino acids (AA) had been artificially modified (15, 65, 67, 89, 99, 107, 110, 118, 134, 138, 147, 169–171). The SNV derived peptides were neoantigen, or a new AA sequence that forms on cancer cells when somatic mutations occur in tumor DNA sequences (40, 44, 58, 62, 67, 70, 80, 83, 84, 106, 114, 117, 123, 131, 143, 152, 156, 159, 161, 163, 165).

We found that more than half the peptides (51.4%) used in cancer vaccine preparation were overexpressed in the targeted tumor cells (**Figure 1C**). We also found that Montanide ISA-51 was the most widely used adjuvant in cancer vaccines. IL-2 was the most widely used cytokine as a vaccine adjuvant in cancer vaccines (**Figure 1D**). Interestingly, most clinical phase peptides are focused on the HLA class I alleles, especially the A02 and A24, as shown in **Figure 1E**. In addition, we noted that more than half of peptide-based cancer vaccines were injected weekly (53.9%, **Figure 1F**) and more than half of the patients had greater than four vaccine injections (**Figure 1G**).

### The prognostic evaluation of anti-tumor effect in clinical trials

After building this library, we wanted to further explore the causes which influence the effectiveness of peptide-based cancer vaccine results. We divided these peptide-based cancer vaccine results into high clinical response (HCR) and low clinical response (LCR) results based on their clinical efficacy and safety (172).

**Figure 2:**
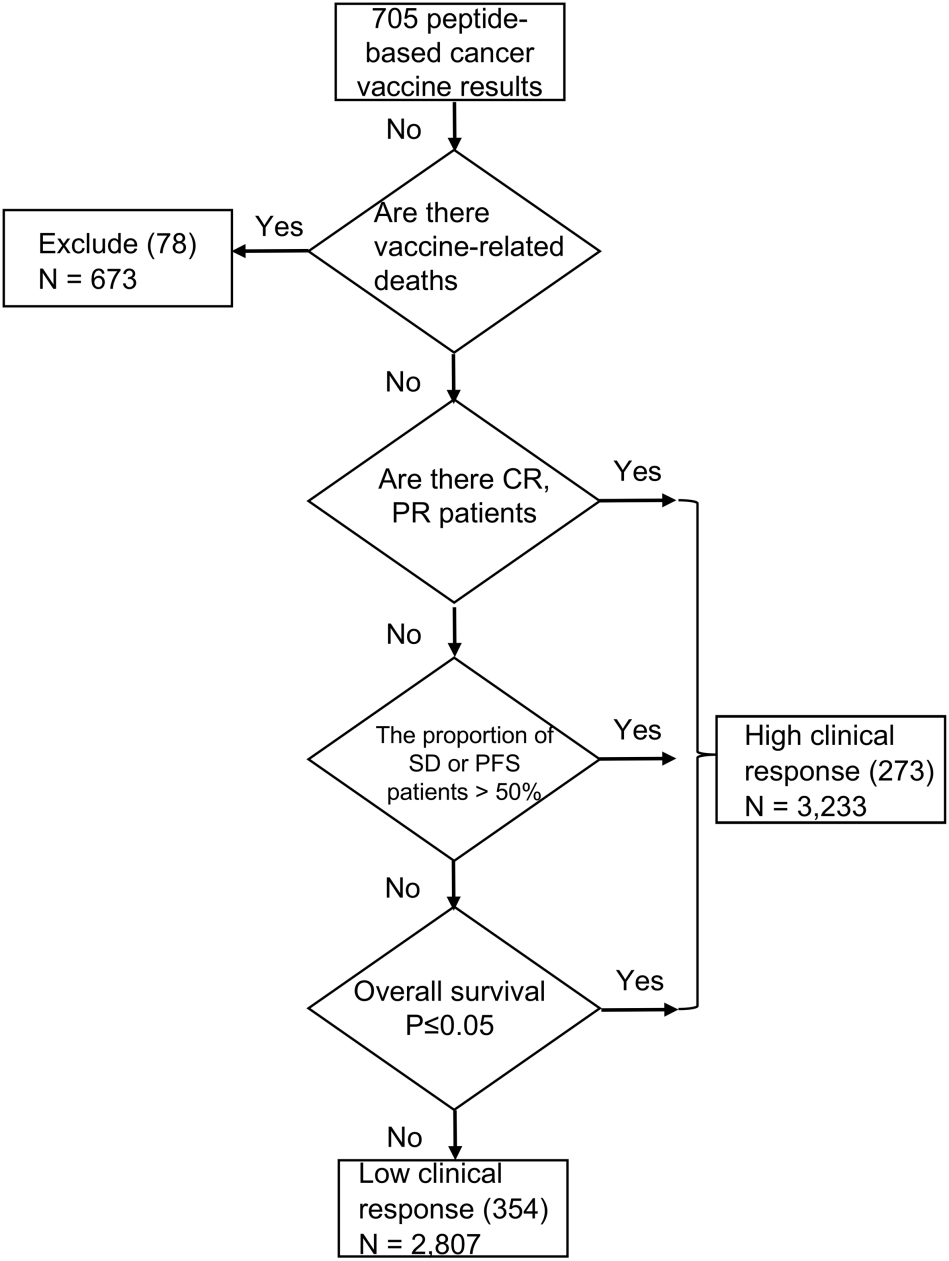
Cancer vaccine criteria and feature comparison. The specific criteria of a clinical treatment response. A total of 78 peptides resulting which involved 673 patients were excluded. 273 high clinical response results (3,233 patients involved) and 354 low clinical response results (2,807 patients involved) were finally included in this study.

The specific classification criteria and basis of a high clinical response were presented in the form of an evolutionary tree, as shown in **Figure 2**. The prerequisite criteria was whether there have been any vaccine-related deaths; if there were vaccine-related deaths, it was excluded from this study. A total of 78 peptides which involved 673 patients were excluded from this study. We next examined whether patients in the best objective response (complete or partial response, according to modified World Health Organization criteria) had been reported (172–174). Due to the complex tumor microenvironment and vaccine technology limitations, it is difficult to achieve a complete response with vaccine therapy; therefore, the best objective response indicates that the clinical response of the vaccine therapy is high (175). We also looked at cases where there were no best objective response patients, investigating whether more than 50% of patients achieved stable disease (SD) or progression-free survival (PFS) if the previous conditions were not met. If more than half of the patients achieved SD or PFS, we took this as an indication that the cancer vaccine was effective (174, 176). Finally, we compared whether the overall survival time was significantly longer in the vaccine group than in the control group; a significantly longer overall survival time in the vaccine group could also indicate that the vaccine worked well (177, 178). If none of these progressive conditions were met, then the clinical result was classified as a low clinical response result. The clinical efficacy evaluation was performed using the immune-related response criteria (irRC) (179) and response evaluation criteria in solid tumors (RECIST 1.1) standard criteria (180). Toxicities were reported using the World Health Organization grading system (181). In total, 273 high clinical response results (3,233 patients involved) and 354 low clinical response results (2,807 patients involved) were included in this study (**Figure 2**).

To further investigate which factors influence the efficacy of cancer vaccines, we specifically explored the types of peptides, HLA alleles, adjuvants, and treatment regimens (injection interval and injection times) in the high and low clinical response groups. Further details are provided in the following sections.

### Modified and tumor overexpressed peptides could be suitably selected for cancer vaccines

**Figure 3:**
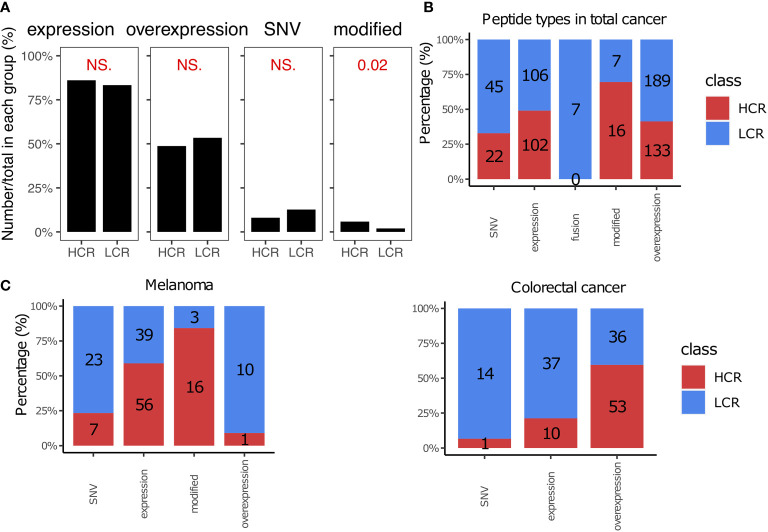
Peptide types in high and low clinical response results. **(A)** Comparison of peptide types between high clinical response (HCR) results and low clinical response (LCR) results. The distribution of peptide types in HCR and LCR results. **(B)** The distribution of peptide types in HCR and LCR results. **(C)** The distribution of peptide types in HCR and LCR results in melanoma and colorectal cancer, respectively. P-values were calculated using two-sided Wilcoxon rank-sum tests. NS., not significant.

Cancer vaccines face a number of challenges, including finding suitable sources of peptides that work best *in vivo*. The peptides were divided into five categories based on the origin of peptides (182, 183): the tumor expressed peptides, tumor overexpressed peptides, fusion peptides, modified peptides, and single-nucleotide variant (SNV) peptides. We summarized their distribution in the results from high and low clinical responses (**Figure 3**). As shown in **Figure 3A**, most of the peptides in the library are expressed or overexpressed in cancer. The overexpressed peptides achieved many of the high clinical response results (41.3%), especially in colorectal cancer (59.6% *vs*. 40.4% in high *vs*. low clinical response results, **Figures 3A**, **C**). The fusion cancer vaccines were not as efficacious in clinical trials, as they did not lead to any high clinical responses (**Figure 3A**). However, modified peptides, in which the amino acids (AA) were artificially modified, appeared to be the most suitable method for cancer vaccine (69.6% of modified peptides achieved high clinical response results), especially for melanoma immunotherapy (84.2%, **Figures 3B**, **C**). In this study, we also listed the top 8 most frequent peptide gene names and the top 18 most commonly used peptides, as shown in **Figure S1**.

### HLA class II peptide-based cancer vaccines could achieve high clinical response results

**Figure 4:**
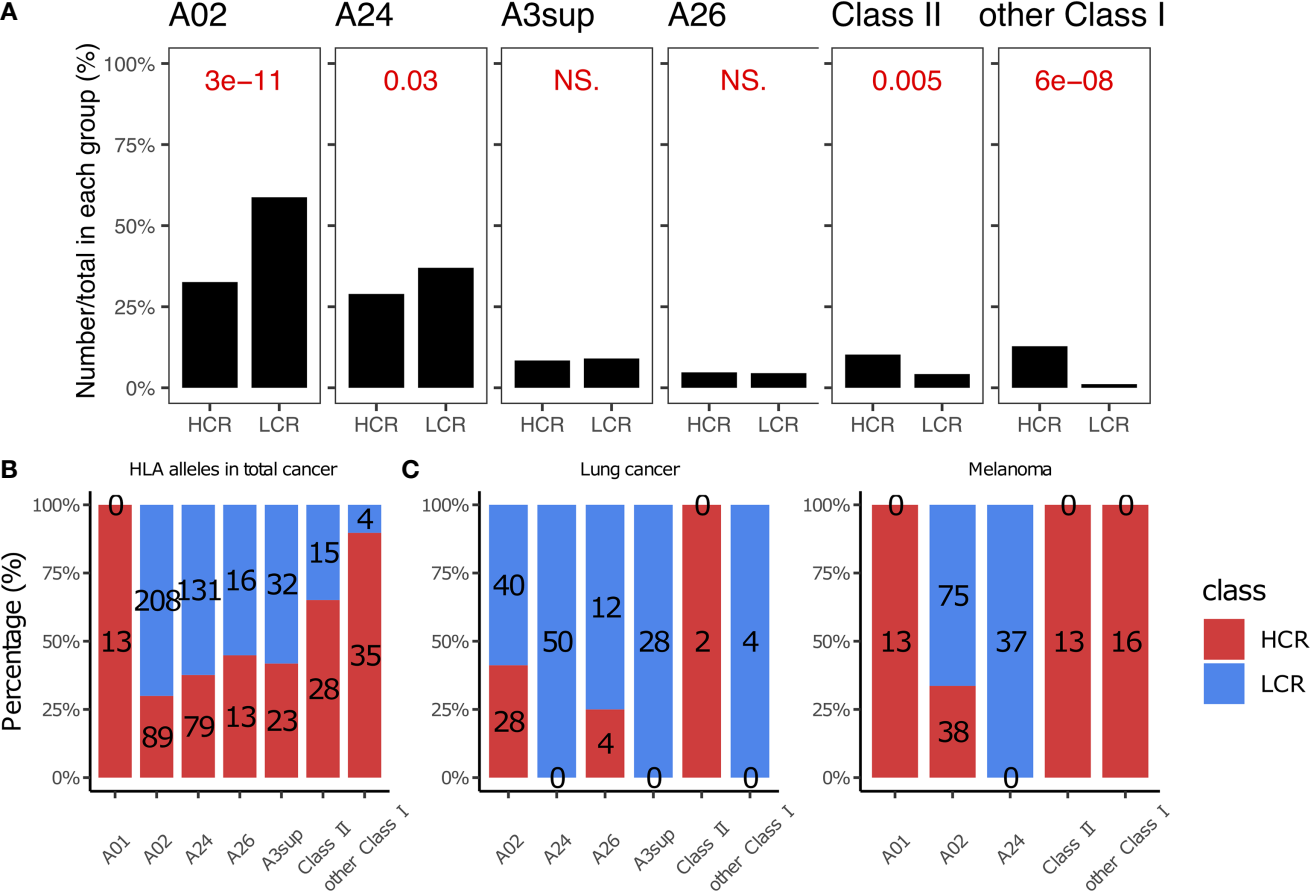
HLA alleles in high and low clinical response results. **(A)** Comparison of HLA alleles between HCR and LCR results. **(B)** The distribution of HLA alleles in HCR and LCR results. **(C)** The distribution of HLA alleles in HCR and LCR results in melanoma and lung cancer, respectively. P-values were calculated using two-sided Wilcoxon rank-sum tests. NS, not significant.

The cytotoxic T cell (CTL) epitope peptides were restricted with HLA alleles when they were prepared as cancer vaccines (102, 184–186). Previous studies have reported that an accurate HLA allele is a key factor in successful cancer vaccinations (186, 187). More than 90% of cancer vaccines in our library are typed for HLA Class I alleles (**Figure 4A**), with the most common being HLA- A02, A24, A3sup, A26, and A01. This is generally consistent with the proportional rank of these alleles in the population (188, 189). We noticed that patients with melanoma achieved more high clinical response results when using peptides typed for HLA-A01 and HLA Class II. For example, all peptides with HLA-A01 alleles achieved high clinical response results in melanoma (13 *vs*. 0, **Figures 4B**, **C**), although this is limited by the sample size and we may need more data to verify whether a similar trend exists in other cancer types (**Figure S4**). The peptides with HLA Class II alleles also achieved more high clinical response results (28 *vs*. 15, P = 0.0049, **Figures 4A**, **B**), especially for melanoma and lung cancer patients (**Figure 4C**). HLA Class II alleles are highly associated with the CD4+ T cells; CD4+ T cells primarily mediate anti-tumor immunity by providing help for CD8+ CTL and antibody responses, as well as *via* secretion of effector cytokines such as interferon-γ (IFNγ) and tumor necrosis factor-α (TNFα). Under specific contexts, they can also mediate anti-tumor immunity *via* direct cytotoxicity against tumor cells (190–193). Therefore, peptide epitopes targeting HLA Class II could be more likely to achieve high clinical response results.

### Montanide ISA-51 was identified as an effective adjuvant in the treatment of cancer vaccines, especially for breast and colorectal cancers

An adjuvant is an ingredient that can help create a stronger immune response in patients receiving the vaccine (194, 195). Many cancer vaccines use adjuvants to enhance therapeutic efficacy.

**Figure 5:**
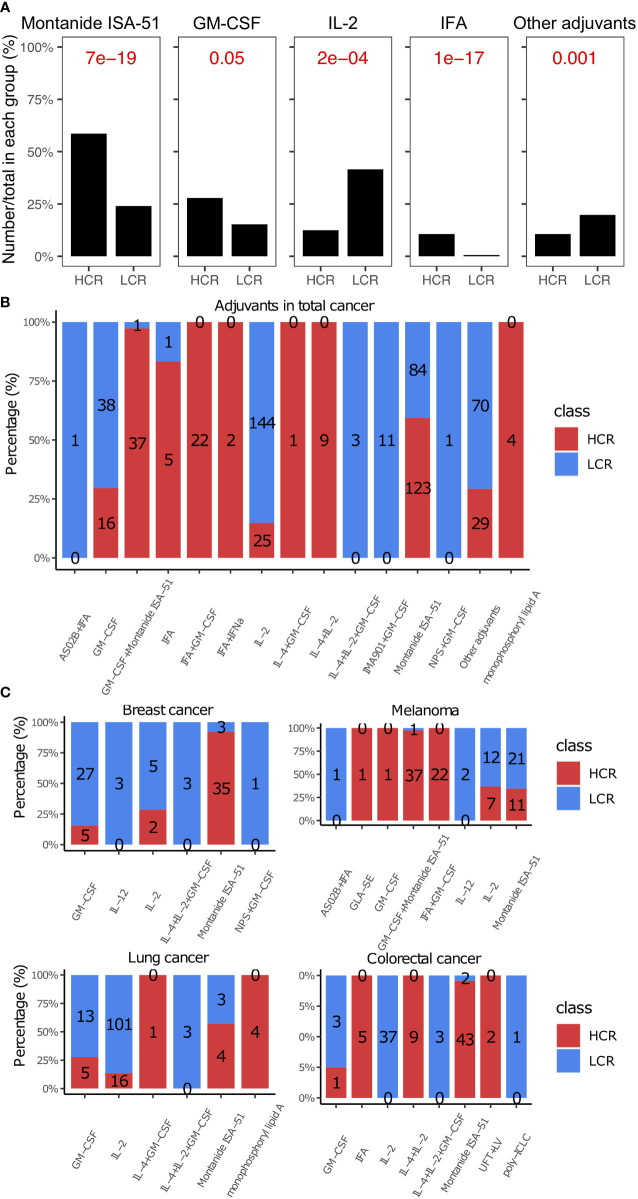
Adjuvants in high and low clinical response results. **(A)** Comparison of adjuvants between HCR and LCR results. **(B)** The distribution of adjuvants in HCR and LCR results. **(C)** The distribution of adjuvants in HCR and LCR results in four main cancer types (breast cancer, melanoma, lung cancer, and colorectal cancer). P-values were calculated using two-sided Wilcoxon rank-sum tests.

We found that Montanide ISA-51 and Granulocyte-macrophage colony-stimulating factor (GM-CSF) were the two most widely used adjuvants in cancer vaccines (**Figure 5A**), and they also appeared most frequently in high clinical response results (P = 6.83e-19 and 0.045, respectively, **Figures 5A**, **B**). Montanide ISA-51, in particular, was the most frequently used adjuvant with high clinical response results (59.4%, **Figure 5B**), especially in breast cancer and colorectal cancer (92.1% and 95.6%, respectively, **Figure 5C**). Cytokines in cancer immunity and immunotherapy, cytokine modulation is necessary for efficacious treatment of experimental neuropathic pain (196). IL-2, IL-4, and IL-12 were the most widely used cytokines as vaccine adjuvants, especially IL-2, which was used as an immune adjuvant in many kinds of cancer types, such as lung cancer, colorectal cancer, and melanoma (**Figure 5C**). However, IL-2, IL-4, and IL-12 are not very effective when used alone in peptide-based cancer vaccines (**Figures 5B**, **C**).

### Treatment regimens with weekly intervals and greater than four injections could be more likely to achieve a better clinical response

The type of treatment regimen is also one of the major challenges affecting the effectiveness of cancer vaccine therapy (32, 197, 198). We evaluated the influences of treatment regimens on cancer vaccine efficacy.

**Figure 6:**
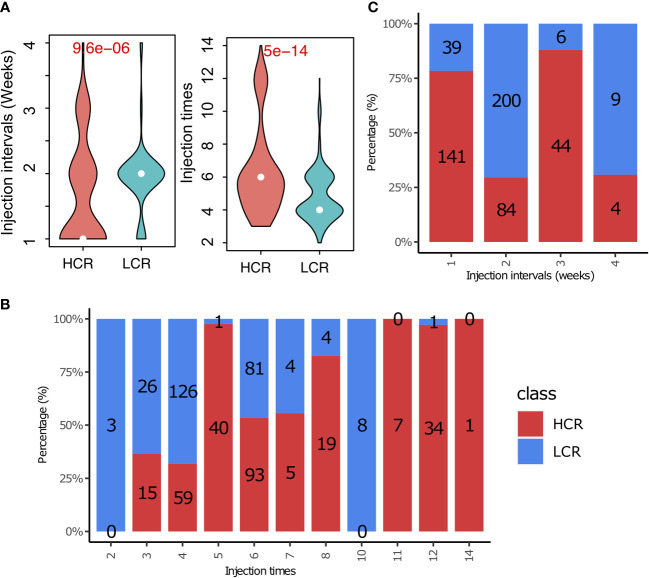
Treatment regimens play an important role in cancer immunotherapy. **(A)** The distribution of treatment regimens (injection interval) in HCR and LCR results. **(B)** The distribution of treatment regimens (injection times) in HCR and LCR results. **(C)** Comparison of treatment regimens (injection interval and injection times) between HCR and LCR results. P-values were calculated using two-sided Wilcoxon rank-sum tests.

Our study found a significant difference in the vaccine injection intervals between the HCR and LCR results (**Figure 6A**, left). Treatment regimens with shorter vaccine injection intervals yielded more high clinical response results. The number of high clinical response results decreases from 141 to 4 with the increase in vaccine injection intervals, implying that shorter vaccine injection intervals (weeks) may be more favorable for patients to achieve high clinical response results, as shown in **Figure 6A**. We also found that the number of vaccine injections associated with high clinical response results was significantly higher than that of the low clinical response results (**Figure 6A**, right). Patients with greater than four vaccination injections achieved more high clinical response results (**Figure 6C**). We also found similar results in main cancer types, such as breast cancer, melanoma, colorectal cancer, and lung cancer. The details are shown in the supplement materials [injection interval (**Figure S2A**) and injection times (**Figure S2B**), respectively].

### Generation of a random forest model from clinical responses for cancer vaccines

**Figure 7:**
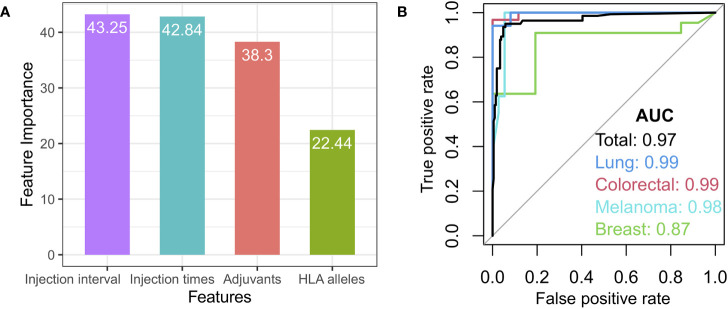
Features selection and model. **(A)** The variable importance for the selected features, such as injection interval, injection times, adjuvant types, and HLA alleles. **(B)** Receiver Operating Characteristic (ROC) curve for the total test set (black) and independent breast cancer (green), melanoma (light blue), lung cancer (blue), and colorectal cancer datasets (red).

Given that some redundant or irrelevant features in the new data set may exert an influence on the classifying effects of a machine learning model, the importance value of cancer vaccine features was first calculated by means of a Random Forest algorithm, followed by the selection of the optimal features based on each feature’s importance (**Figure 7A** and **Supplemental Figure S3**). From the methodological perspective of feature selection, the random forest is a kind of embedded feature selector that can automatically produce the relative importance of features during the model training process. We investigated the relative importance of multiple features, such as the peptide types, adjuvant, HLA alleles, tumor stages, chemotherapy, and treatment regimens, in cancer vaccines. Four features were chosen for the random forest-based modeling in this study: injection interval, injection times, adjuvant types, and HLA alleles (**Figure 7A**).

For the random forest-based modeling, the library cohort with known high or low clinical response was randomly split into a training set (50% randomly selected samples) and a test set (50% randomly selected samples) according to the cancer types. The merged training set was used as the training set and the merged test set as the test set. The model was trained on the training set and tested on the test set, with 1,000 repeated nested 5-fold cross-validation. The model achieved a high area under the curve (AUC) of 0.97 sensitivity on the test set (**Figure 7B**, black curve). The prediction model’s performance was first assessed in four independent cancer type cohorts (breast cancer, melanoma, lung cancer, and colorectal cancer) with the equilibrium of class distribution and balanced data. Our model also achieved AUCs of 0.87, 0.99, 0.99, and 0.98 for independent breast cancer, melanoma, lung cancer, and colorectal cancer datasets, respectively, demonstrating that our model could predict their vaccine responses from the features we selected (**Figure 7B**). In addition, the prediction model was also evaluated from the perspectives of the average precision score and precision-recall (AUPRC) (**Figure S3D**).

Random forest yields high discriminative performance in cancer vaccine clinical response prediction. Thus, the model could be helpful in identifying cancer vaccines with high clinical responses.

## Discussion

Immunotherapies such as peptide-based cancer vaccines have proven to be effective in enhancing the immune response in cancer patients to fight cancer cells. Cancer vaccines that specifically target high expression of gp100 in melanoma have already been approved (199). However, one of the key factors limiting the application of immunotherapy is how to rationally design a peptide-based cancer vaccine that generates an anti-tumor immune response leading to the effective killing of tumor cells (200–202). The goal of our study is to determine the key criteria for cancer vaccines that may lead to better clinical outcomes.

We collected T cell epitopes from several databases that had been applied to clinical studies to construct *in silico* a library of peptide-based cancer vaccines. These candidate T cell epitopes could activate CD8+ or CD4+ T cells to induce cytotoxicity for tumor cells. The selected peptide epitopes could be used as cancer vaccines or as target antigens for adoptive cell therapy of DC, CTL, TCR-T, and CAR-T cells. To find the associations which influence treatment effectiveness of cancer vaccines, several critical factors, including types of peptides, HLA alleles, adjuvants, and treatment regimens, were analyzed in patients with high and low clinical responses.

We found that studies often chose tumor expressed or overexpressed peptides to prepare cancer vaccines (**Figures 1**, **3**). However, modified peptides in which the amino acids (AAs) always were artificially modified, could be more suitable for cancer vaccines, especially for melanoma immunotherapy (65, 67, 79, 99, 110, 118, 147) (**Figure 3**). The reason modified peptides are more effective may be because the AAs of modified peptides are altered and these modified peptides will be treated as exogenous peptides, which are more easily recognized by T cells and therefore more immunogenic (12, 203–205). Alternatively, the genes of these modified peptides are always expressed or overexpressed in tumor cells (**Figure S1**), and their comparative wild-type peptides are usually known to have binding affinities for certain HLA alleles (206–208). Based on this information, we could make targeted modifications to the modified peptides, further enhancing the affinity of modified peptides for pMHC, potentially making modified peptides more immunogenic and therapeutically effective. However, it is possible that modified peptides are also expressed in normal cells, which could lead to uncertain side effects (205, 208).

Based on our study, Montanide ISA-51, in particular, was the most frequently used adjuvant with high clinical response results (59.4%, P = 1.3e-17, **Figure 5**), especially in breast cancer and colorectal cancer (92.1% and 95.6%, respectively, as shown in **Figure 5A, C**). Montanide ISA-51 can enhance antigen-specific antibody titers and cytotoxic T-lymphocyte responses. Doorn et al. reported that a proper mixture of peptide epitopes and Montanide ISA-51 could help effectively avoid or mitigate adverse events (209, 210). Because cytokine modulation is necessary for efficacious treatment of experimental neuropathic pain (196). Many cytokines, such as IL-2, IL-4, and IL-12, were widely used cytokines as vaccine adjuvants. IL-2 in particular was used as an immune adjuvant in many cancer types, such as lung cancer, colorectal cancer, and melanoma. However, as vaccination adjuvants, these cytokines are less efficient than Montanide ISA-51 or GM-CSF (**Figure 5**). There are several possible explanations for this. One explanation is that cytokines are susceptible to degradation due to their short half-life (211, 212). Another explanation could be that the main role of adjuvants is to activate the immune system and activate effector T cells (194). If we use IL-2, IL-4, and IL-12 as the adjuvant in cancer vaccine therapy, we need them to activate effector T cells. However, IL-2 has a higher affinity and stimulatory effect on regulatory T cells (Tregs). Therefore, IL-2 will activate Treg cells along with effector T cells. Tregs are a specialized subpopulation of T cells that act to suppress the immune response. Thus, it is difficult to achieve a high clinical response with IL-2 alone. IL-4 is a cytokine that induces the differentiation of naive helper T cells (Th0 cells) to Th2 cells, thereby inducing immunosuppression (213). Therefore, IL-4 generally does not have a good antitumor effect in cancer vaccines as an adjuvant alone. IL-12 is an interleukin that is naturally produced by dendritic cells (214). It has a strong anti-tumor therapeutic effect, but IL-12 is difficult to use in molecular therapy alone (215). Thus, IL-2, IL-4, and IL-12 are not very effective when used alone in peptide-based cancer vaccines. Antigen-specific specificity is important in cancer vaccinations. However, antigen-specific cytokines do not exist; cytokines can activate many non-specific or tumor growth promoting pathways, which is ineffective and unhelpful for the specificity of a cancer vaccine (216, 217). IL-2, IL-4, and IL-12 may need to be modified or combined with other adjuvants, such as Montanide ISA-51 or GM-CSF in peptide-based cancer vaccine therapies to increase effectiveness.

In addition to these cancer vaccines that utilized various adjuvants, we also observed that many cases did not use any adjuvants in cancer vaccine preparation, which could be a factor leading to the poor clinical outcomes of many peptide vaccinations in clinical trials (38, 41, 49, 53, 68, 72, 74, 81, 82, 86, 95, 98, 110, 117, 127, 131, 143, 151, 160, 218–221)

The treatment regimen also plays an important role in the therapeutic efficacy of cancer vaccines. The treatment regimens with weekly injection intervals and greater than four vaccination injections were more likely to achieve a high clinical response (**Figure 6**). Short vaccine injection intervals and multiple vaccinations could continually activate the immune system, ensuring that there are enough cytotoxic T cells to kill tumor cells, enhancing the tumor-killing effect and making it easier to get a high clinical response (222). However, many of the available treatment regimens did not use shorter vaccine intervals and more frequent injections. This could be because many of the patients who participated in the cancer vaccine clinical trials had gone through various conventional treatments, and many of them also had stage III or stage IV tumors. As a result, they were already in a weakened condition. Shortened vaccination intervals and an increased frequency could lead to stronger side effects that would be difficult for these weaker patients to tolerate. Therefore, shorter intervals and more frequent injections may be more appropriate for patients with early-stage tumors, while relatively frail patients could have longer injection intervals, but no less than four injections are recommended.

Finally, we investigated the correlation between multiple features, such as the peptide types, adjuvant, HLA alleles, and treatment regimens, in cancer vaccines, and we built a random forest model to distinguish the peptide-based cancer vaccines with high clinical responses (**Figures 7**, **S3**). In addition, we also investigated the effect of the tumor stages and chemotherapy, which could reflect the patient’s health condition and medical treatment prior to vaccination on the model prediction. We summarized their distribution in the results from the high and low clinical responses (**Figures S4A**, **B**). The relative importance of the tumor stages and chemotherapy are weaker than the peptide types, adjuvant, HLA alleles, and treatment regimens in this study (**Figure S4C**). The prediction accuracy of our model was also not improved by introducing them (**Figure S4D**). Possible causes for this are that many clinical trials included patients with different tumor stages, and some others omitted patients’ tumor stages information. There is no significant difference between the chemotherapy group and the no or unknown chemotherapy group (**Figure S4B**). The cause could be that many of the cancer vaccine trials included patients who had undergone conventional medical treatments, which included but are not limited to chemotherapy. Moreover, some clinical trials excluded patients who had received chemotherapy (41, 45) or required patients to wait a period of time after chemotherapy before participating in cancer vaccine therapy to eliminate and minimize the impact of chemotherapy on vaccine therapy (49, 64, 74, 133, 220). Therefore, we think that the tumor stage and chemotherapy may have less impact on the improvement of prediction accuracy (**Figure S4D**).

We found a combination of the modified peptides, Montanide ISA-51, a short interval between vaccine injections, and multiple vaccinations could be helpful in effectively activating the immune system to kill tumors, enhancing the tumor-killing effect, and resulting in high clinical responses.

The severe acute respiratory syndrome coronavirus 2 (SARS-CoV-2) virus is prone to specific mutations that alter viral surface peptide epitopes, making the virus more susceptible to immune escape (223). Peptide-based tumor vaccine research has also contributed to the development of COVID-19 vaccines targeting COVID-19-specific peptide epitopes (224, 225).

There are several limitations noted in this study. First, there are no prior studies that quantitatively distinguish between high or low results in cancer vaccine clinical trials; it is possible that we missed information in classifying a high or low response result. Next, due to lack of sufficient data for a single tumor type, we could not directly explore the difference in each cancer type between the high- and low- clinical response groups.

In addition, the effect of the tumor mutation burden, the dose of the cancer vaccine, and the specific method of injection, such as subcutaneous, intranasal, intravenous, and transdermal, may also need to be further explored in the future studies. Lastly, the effect of coupling multiple features on cancer vaccine efficacy is complex and was not investigated in depth in this study. Thus, future studies can explore further the effects of coupling multiple features on cancer vaccine efficacy based on a larger cohort.

Altogether, we presented CancerVaccine, a peptide-based cancer vaccine library that stored and aggregated the results of peptide-based cancer vaccines and their clinical attributes. CancerVaccine can be accessed at https://peptidecancervaccine.weebly.com/. We demonstrated that CancerVaccine is a versatile resource that can be used to screen for useful peptides epitopes and aid in the design of new cancer vaccines. Our study describes a design strategy in peptide vaccination treatment relating to the appropriate types of peptides, suitable adjuvants, matched HLA alleles, and suitable treatment regimens. Furthermore, we developed a high AUC machine learning model, which could be helpful in identifying peptide-based cancer vaccines with high clinical responses. The results of this study could impact future exploration of vaccine designs, taking into consideration identifying suitable peptide antigens and treatment conditions for cancer and personalized immunotherapy.

## Data availability statement

The original contributions presented in the study are included in the article/**Supplementary Material**. Further inquiries can be directed to the corresponding author/s.

## Author contributions

C-CC and CJ conceived the project. CJ, JL, WZ, ZZ, and GL prepared and analyzed the database. CJ and C-CC, evaluated the conclusions, wrote the manuscript. CJ, C-CC, WH, BL, and XZ reviewed and revised the content. All authors read and approved the final manuscript. All authors contributed to the article and approved the submitted version.

## Funding

This work is supported by the Cancer Prevention Research Institute of Texas (CPRIT) (RR180061).

## Acknowledgments

We would like to give special thanks to Chao Cheng and other members of the Chao Cheng lab for their valuable discussions and critical feedback. We especially thank Aude Angelini, Xiaoshi Zhang, Si Qiu, Wenhui Li, and Xiuying Li for their valuable suggestions.

## Conflict of interest

Author C-CC was employed by the company Biomap, Inc. Authors WZ, ZZ, GL, XZ and BL are employed by the company BGI-Shenzhen.

The remaining authors declare that the research was conducted in the absence of any commercial or financial relationships that could be construed as a potential conflict of interest.

## Publisher’s note

All claims expressed in this article are solely those of the authors and do not necessarily represent those of their affiliated organizations, or those of the publisher, the editors and the reviewers. Any product that may be evaluated in this article, or claim that may be made by its manufacturer, is not guaranteed or endorsed by the publisher.
