# Supplementary material for: Potential association factors for developing effective peptide-based cancer vaccines
Source: Front Immunol. 2022 Jul 27;13:931612. doi: 10.3389/fimmu.2022.931612 (PMC9364268; doi:10.3389/fimmu.2022.931612)
Supplement: Supplementary file 1 [file DataSheet_1.docx]

**Supplementary Figures**

**Supplementary Figure 1. The distribution of top genes and peptides.** A. The top8 genes used in HCR and LCR results. B. The top8 genes used in HCR and LCR results in four main cancer types (breast cancer, melanoma, lung cancer, and colorectal cancer). C. The top 18 peptide antigens used in the clinical treatments of peptide vaccination.

**Supplementary Figure 2. The distribution of treatment regimens (injection interval and injection times) in HCR and LCR results in four main cancer types.** A. The distribution of treatment regimens (injection interval) in HCR and LCR results in four main cancer types (breast cancer, melanoma, lung cancer, and colorectal cancer). B. The distribution of treatment regimens (injection times) in HCR and LCR results in four main cancer types (breast cancer, melanoma, lung cancer, and colorectal cancer).

**Supplemental Figure 3. Features selection.** A. Confirmation of the type of variables that we have in the data. There are no missing values in the data. B. The categorical variables in the dataset. In our dataset, the levels of all categorical variables appeared correct. C. Accuracy of the model based on the number of features (the blue dot represents the optimal solution, 4 features are selected). D. Area under a precision-recall curve (AUPRC) for the total test set (black) and independent breast cancer (green), melanoma (light blue), lung cancer (blue), and colorectal cancer datasets (red).

**Supplemental Figure 4. The effect of tumor stage and chemotherapy features on the clinical response and model prediction.** A. The distribution of tumor stages in high and low clinical response results. B. The distribution of chemotherapy in high and low clinical response results. C. The variable importance for the selected features, such as injection interval, injection times, adjuvant types, HLA alleles, stages, and chemotherapy. D. Receiver Operating Characteristic (ROC) curve for the total test set (black) and independent breast cancer (green), melanoma (light blue), lung cancer (blue), and colorectal cancer datasets (red).
